# Bioadhesive Microcarriers Encapsulated with IL‐27 High Expressive MSC Extracellular Vesicles for Inflammatory Bowel Disease Treatment

**DOI:** 10.1002/advs.202303349

**Published:** 2023-09-27

**Authors:** Min Nie, Danqing Huang, Guopu Chen, Yuanjin Zhao, Lingyun Sun

**Affiliations:** ^1^ Department of Rheumatology and Immunology Nanjing Drum Tower Hospital Affiliated Hospital of Medical School Nanjing University Nanjing 210002 China; ^2^ State Key Laboratory of Bioelectronics School of Biological Science and Medical Engineering Southeast University Nanjing 210096 China; ^3^ Department of Rheumatology and Immunology The First Affiliated Hospital of Anhui Medical University Hefei 230000 China

**Keywords:** adhesion, extracellular vesicle, IL‐27, inflammatory bowel disease, mesenchymal stem cell, microcarrier

## Abstract

Mesenchymal stem cell (MSC) therapy is a promising candidate for inflammatory bowel disease (IBD) treatment, while overcoming the limitations of naive seeding cells function and realizing efficient intestinal targeting remains a challenge. Here, a bioadhesive microparticle carrying interleukin‐27 (IL‐27) MSC‐derived extracellular vesicles (MSC^IL‐27^ EVs) is developed to treat IBD. The MSC^IL‐27^ EVs prepared through lentivirus‐mediated gene transfection technology show ideal anti‐inflammatory and damage repair function. By encapsulating MSC^IL‐27^ EVs into dopamine methacrylamide‐modified hydrogel, a bioadhesive EVs microcarrier via microfluidic technology is fabricated. The resultant microcarriers exhibit ideal MSC^IL‐27^ EVs sustained release effect and effective wet adhesion property. Furthermore, the therapeutic potential of MSC^IL‐27^ EVs‐loaded microcarriers in treating IBD is demonstrated. Through giving IBD rats a rectal administration, it is found that the microcarriers can firmly anchor to the surface of colon, reduce the inflammatory response, and repair the damaged barrier. Therefore, the bioadhesive MSC^IL‐27^ EVs‐loaded microcarriers provide a promising strategy for the biomedical application of MSC‐derived EVs, and broaden the clinical potential of MSC therapy.

## Graphical abstract

A bioadhesive extracellar vehicles (EVs) microcarrier is fabricated by encapsulating MSC^IL‐27^ EVs into dopamine methacrylamide‐modified hydrogel via microfluidic technology for inflammatory bowel disease treatment. Microcarriers are demonstrated to reduce the inflammatory response and repair the damaged barrier. The adhesive microcarrier may be a new bioengineered strategy for the biomedical application of multifunctional EVs and associated therapeutics.

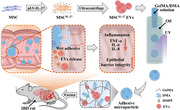

## 1 Introduction

Inflammatory bowel disease (IBD) is a kind of autoimmune disease involving both small and large intestines.^[^ ^1^, ^2^ ^]^ Clinically, anti‐inflammatory and immunosuppressant drugs show promising efficacy in treating IBD.^[^ ^3^, ^4^, ^5^, ^6^, ^7^, ^8^ ^]^ Considering the drug resistance and adverse reactions caused by long‐term use and high dosage, more efficient and targeted therapeutic treatments to avoid adverse inflammatory immune responses have been developed recently.^[^ ^9^, ^10^, ^11^, ^12^ ^]^ Take mesenchymal stem cell (MSC) therapy for instance, the MSCs have great immunosuppressive properties to correct inflammatory disorders.^[^ ^13^, ^14^, ^15^, ^16^, ^17^ ^]^ Preclinical and clinical studies have verified that the MSCs can mediate T cells activation and transform the function of dendritic cells and macrophages.^[^ ^16^, ^18^, ^19^, ^20^, ^21^, ^22^, ^23^ ^]^ However, MSC therapy has not achieved its desired clinical potential in IBD treatment because the therapeutic activity of naive seeding cells is limited.^[^ ^24^, ^25^, ^26^, ^27^, ^28^ ^]^ In addition, existing intravenous injection strategy is generally systemic with several limitations such as poor targeting and fast metabolism.^[^ ^29^, ^30^ ^]^ Therefore, the development of an effective delivery system to extend the application of MSC therapy in IBD treatment is still highly anticipated.

**Figure 1:**
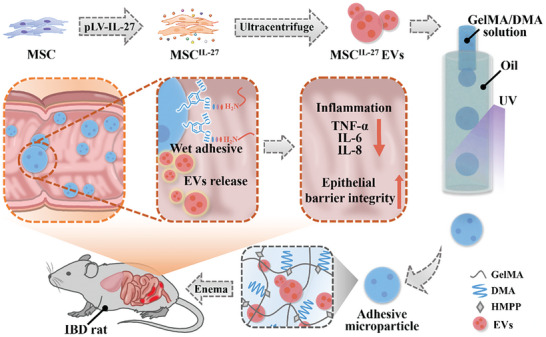
Schematics of the fabrication and application of the wet‐adhesive microparticles with MSC^IL‐27^ EVs loaded for IBD treatment. The MSC^IL‐27^ EVs prepared via lentivirus‐mediated gene transfection technology and ultracentrifuge were encapsulated into DMA‐modified GelMA through microfluidic method. The MSC^IL‐27^ EVs‐loaded microparticles were applied through rectal administration to treat IBD rat.

Herein, we present a novel bioadhesive microcarriers delivery system encapsulated with interleukin‐27 (IL‐27) highly expressive MSC extracellular vehicles (EVs) to treat IBD through rectal administration, as schemed in **Figure** **1**. IL‐27 is a type I cytokine with established functions in the occurrence and progression of infectious diseases, autoimmune diseases, and cancers.^[^ ^31^, ^32^, ^33^, ^34^, ^35^, ^36^, ^37^ ^]^ It has also been identified as a candidate target gene for treating IBD.^[^ ^38^, ^39^ ^]^ Numerous studies have demonstrated that IL‐27 can promote intestinal epithelial barrier integrity and suppress neutrophil function in IBD mouse models.^[^ ^40^, ^41^, ^42^ ^]^ Compared to MSCs, EVs display higher plasticity and maneuverability in biomedical applications.^[^ ^43^, ^44^, ^45^ ^]^ For example, by encapsulating EVs into biocompatible and biomodifiable microcarriers, it is anticipated to realize the sustained release of therapeutic cytokines.^[^ ^46^, ^47^ ^]^ In addition, introducing adhesive chemical molecules, such as dopamine methacrylamide (DMA), into the EVs delivery system might allow the targeted adhesion to the lesion sites in intestines.^[^ ^48^, ^49^ ^]^ Therefore, it is conceivable that the integration of highly expressed IL‐27, MSC derived EVs, and adhesive microcarriers would form an unprecedented specific targeting delivery system for IBD treatment.

In this paper, we fabricated the desired bioadhesive microparticle encapsulated with EVs high‐expressing IL‐27 (MSC^IL‐27^ EVs) to treat IBD. The MSC^IL‐27^ EVs were derived from lentivirus‐mediated gene transfected MSCs. It was found that our MSC^IL‐27^ EVs can significantly inhibit inflammation and ameliorate the intestinal epithelial barrier damage. Subsequently, by employing microfluidics to encapsulate the MSC^IL‐27^ EVs and cross‐linking biocompatible hydrogel gelatin methacrylate (GelMA) and bioadhesive molecule DMA, the prepared microparticles showed desired biostability and MSC^IL‐27^ EVs loading efficiency. Due to the exposing of 3, 4‐dihydroxy‐L‐phenylalanine (DOPA) in DMA, the strong wet adhesion ability of the microparticles were confirmed through adhesion assay. Based on these features, we have demonstrated that the MSC^IL‐27^ EVs‐loaded microparticles could firmly stick to the inflamed lesion through rectal administration, which not only contributed to the anti‐inflammatory and barrier repairing efficacy but also reduced the side effects caused by the systematic treatment. Therefore, our MSC^IL‐27^ EVs‐loaded adhesive microparticles provided a new bioengineered strategy for the biomedical application of multifunctional MSC derived EVs, as well as expanding the clinical potential of associated therapeutics.

## 2 Results and Discussion

**Figure 2:**
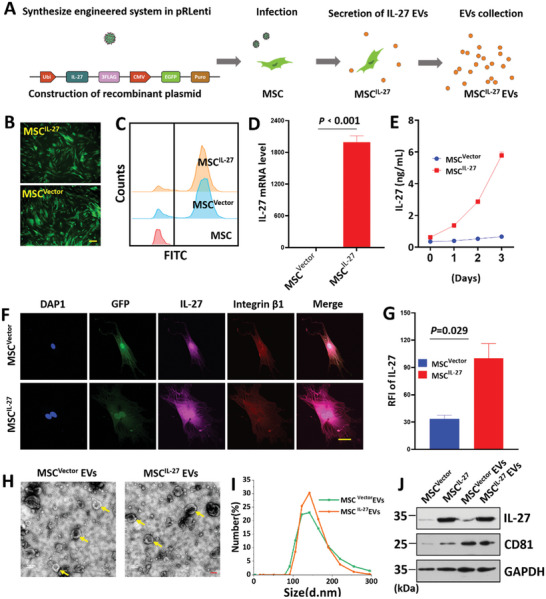
Schematic and characterization of MSC^IL‐27^ EVs. A) Schematic of preparation of MSC^IL‐27^ EVs. B,C) MSC^IL‐27^ and MSC^Vector^ were constructed. Scale bars = 200 µm. D) The expression level of IL‐27 in MSC^Vector^ and MSC^IL‐27^ was tested using Q‐RT‐PCR (*n* = 4), E) ELISA method. F) Confocal laser scanning microscope (CLSM) images of immunofluorescence staining of IL‐27 on MSC^Vector^ and MSC^IL‐27^. Scale bars = 200 µm. G) Corresponding quantification results of relative fluorescence intensity (RFI) of IL‐27 (*n* = 4). H) Representative TEM images of MSC^Vector^ and MSC^IL‐27^ EVs, Scale bars = 100 nm. I) Dynamic light scattering (DLS) showed the size distribution of MSC^Vector^ and MSC^IL‐27^ EVs. J) CD81 and IL‐27 expression on MSC and MSC‐EVs were detected by Western blot. Glyceraldehyde‐3‐phosphate dehydrogenase (GAPDH) was used as loading control.

In this study, MSC^IL‐27^ EVs were prepared through lentivirus‐mediated gene transfection technology and ultracentrifuge. Initially, MSCs were isolated from human umbilical cord tissues. To generate IL‐27 overexpressed MSC, the MSC were infected with lentivirus and screened by puromycin (**Figure** **2**A). The morphology of MSCs was characterized by microscope. As Figure 2B and Figure S1A (Supporting Information)showed, the MSCs displayed spindle shape. The surface markers of MSCs were analyzed by flow cytometry (FCM). MSC^IL‐27^ did not exhibit significant difference in MSC surface markers (Figure S1B,C, Supporting Information), showing that the genetic modification of IL‐27 did not change the morphology and surface markers of MSCs. Meanwhile, FCM was applied to evaluate the transduction efficiency, resulting in over 90% green fluorescent protein (GFP) positive rate (Figure 2C). Subsequently, the levels of IL‐27 were tested by quantitative reverse‐transcription polymerase chain reaction (Q‐RT‐PCR) and enzyme linked immunosorbent assay (ELISA). The results showed that the IL‐27 gene expression was ≈2000‐fold increase compared with the control MSCs (MSC^Vector^) (Figure 2D). As Figure 2E showed, MSC^IL‐27^ secreted ≈6 ng of IL‐27 to the condition medium in 3 days. IL‐27 expression (magenta) and MSC marker integrin *β*1 (red) were detected by immunofluorescence. IL‐27 was expressed on the hole cell (Figure 2F). The MSC‐EVs were then isolated by ultracentrifuge. Transmission electron microscopy (TEM) images displayed spherical pattern of MSC^IL‐27^ EVs with an average diameter ≈150 nm. As shown in Figure 2H,I, there was no obvious difference between MSC^Vector^ and MSC^IL‐27^ EVs. Subsequently, we performed Western blot analyses to measure the EVs‐associated protein CD81. Moreover, we confirmed the high level of IL‐27 in MSC^IL‐27^ and MSC^IL‐27^ EVs (Figure 2J; Figure S2, Supporting Information). These dates indicate that we have obtained IL‐27 overexpressed MSC‐EVs successfully.

**Figure 3:**
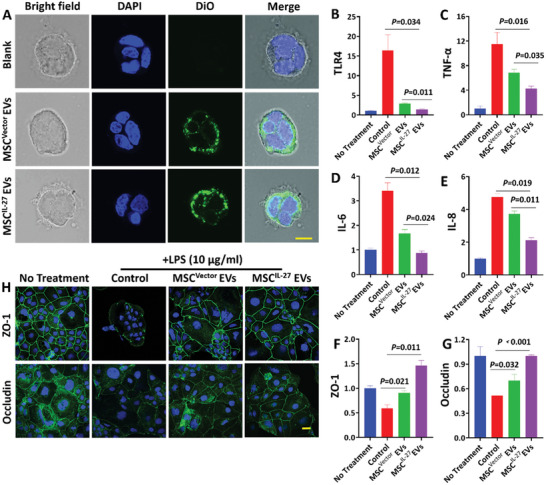
The uptake and function of MSC^IL‐27^ EVs in LPS ‐treated Caco‐2 intestinal barrier model. A) CLSM of Caco‐2 cells incubated with DiO labeled EVs. Scale bars = 100 µm. Effects of EVs on the expressions of TlLR4 B), TNF‐*α* C), IL‐6 D) and IL‐8 E) (*n* = 4). F,G) Effects of EVs on the mRNA levels of ZO‐1 and occludin (*n* = 4). H) ZO‐1 and occludin expressions were analyzed by CLSM. Scale bars = 100 µm.

Next, we analyzed whether the MSC^IL‐27^ EVs were involved in anti‐inflammatory reaction and repaired the damaged intestinal barrier. First, cell uptake of EVs was tested in Caco‐2 (colorectal cancer cell line) and RAW264.7 (macrophage cell line) cells. The EVs were marked with 3,3‐dioctadecyloxacarbocyanine perchlorate (DiO) for 6 h and then cocultured with cells for 24 h. CLSM images showed the appearance of green fluorescence in the cytoplasm (**Figure** **3**A; Figure S3, Supporting Information). Hence, we investigated the anti‐inflammatory and intestinal barrier repair function of MSC^IL‐27^ EVs in lipopolysaccharide (LPS)‐induced intestinal barrier dysfunction. As shown in Figure 3B–E, stimulation of Caco‐2 cells by LPS enhanced Toll‐like receptor‐4 (TLR4) level and accelerated the production of proinflammatory factors including tumor necrosis factor‐*α* (TNF‐*α*), IL‐6, and IL‐8 compared with no treatment group. However, this effect was mitigated by the addition of EVs. Compared with MSC^Vector^ EVs group, MSC^IL‐27^ EVs group decreased more significantly. These results elucidated that MSC^IL‐27^ EVs suppressed the activation of inflammatory cascade in LPS‐treated Caco‐2 cells. Treatment with LPS decreased the levels of zona occludens 1 (ZO‐1) and occludin involved in maintaining intestinal barrier function (Figure 3F,G). Moreover, co‐incubation of EVs modulated the expression of these factors, treatment with MSC^IL‐27^ EVs markedly increased the level of ZO‐1 and occludin compared with MSC^Vector^ EVs. Next, we analyzed the effects of EVs on ZO‐1 and occludin localization based on immunofluorescence. ZO‐1 and occludin were highly expressed and connected without damage in no treatment group. However, ZO‐1 and occludin staining were discontinuous in LPS‐treated Caco‐2 cells (Figure 3H; Figure S4, Supporting Information). These discontinuous pericellular expressions of ZO‐1 and occludin were mitigated by EVs treatment, a strong fluorescence intensity, and more continuous signal pattern in MSC^IL‐27^ EVs group compared with MSC^Vector^ EVs group. As a member of the IL‐12 family, IL‐27 is actively involved in differentiating T cells. Next, we checked the function of MSC^IL‐27^ EVs in T‐cell‐related genes such as IL‐10, IFN (Interferon)‐*γ* and IL‐4. As shown in Figure S5 (Supporting Information), IL‐10 was increase in the groups treated with the EVs compared to no treatment group. Among the EVs treated groups, the effects of MSC^IL‐27^ were more potent on promoting the expression of IL‐10, compared to the MSC^Vector^ group. The expression of IFN‐*γ* and IL‐4 were reduced in the groups treated with the EVs compared to no treatment group. In order to reveal the role of IL‐27 in those processes, we performed RNA sequencing analysis to profile the transcriptome of MSC^Vector^ and MSC^IL‐27^. Compared with the MSC^Vector^ group, there were 121 genes differentially expressed (*p* < 0.001), of which 37 were increased and 84 were reduced (Figure S6, Supporting Information). The Gene Ontology analysis indicated that the differential genes were enriched for functional annotations relating to type I interferon signaling pathway, defense response to other organism, positive regulation of innate immune response, and inflammatory response. All these results clearly indicated that the MSC^IL‐27^ EVs inhibited the inflammatory response, rescued intestinal barrier dysfunction, and regulated the differentiation of T cells.

**Figure 4:**
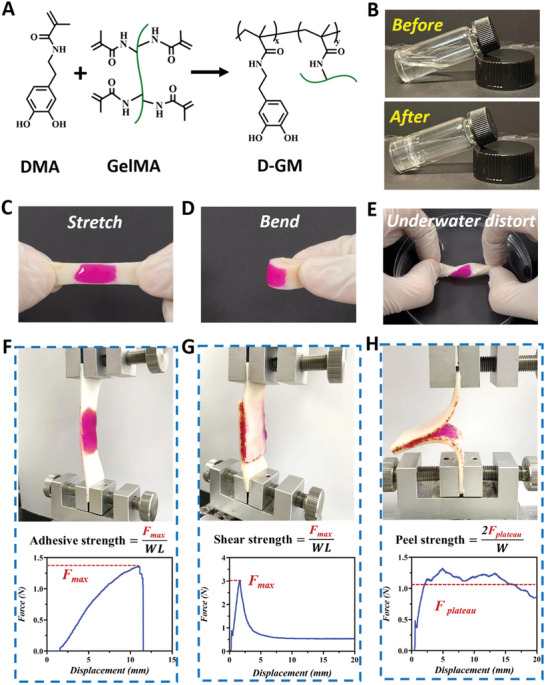
Surface functionalization of hydrogel. A) The preparation of D‐GM hydrogel. B) Optical images of the hydrogel before and after ultraviolet radiation. C,D,E) Mechanical testing of adhesion properties. F,G,H) Setup for measurement of adhesive strength, shear strength, and peel strength. *F*_max_: maximum force; *F*_plateau_: plateau force; in peel test; *W L*: width length.

To expand the application of EVs, we proposed a novel adhesive hydrogel with DMA and GelMA. **Figure** **4A** shows the cross‐linking process of the DMA/GelMA hydrogel (D‐GM). As shown in Figure 4B, GelMA and DMA could form a composite hydrogel under UV radiation. Meanwhile, D‐GM hydrogel adhered to wet tissues under the hydrogen bonds between the catechol and the amino groups. To further study the deformation recovery ability of hydrogel under external forces, the hydrogel was gelatinized on pigskin and colored with rhodamine B. The inset pictures in Figure 4C–E and Figure S7 (Supporting Information) intuitively showed the well‐adhesive and deformation recovery ability of the hydrogel under external forces such as stretching, bending and even distorting under water. Next, we evaluated the in vitro adhesion performance of the hydrogel by adhesive, lap‐shear, and peel tests on porcine skin according to the standard method. As shown in Figure 4F–H, the max adhesive strengths in adhesive, lap‐shear, and peel tests of hydrogel were ≈1.4, 3.0, and 1.1 N mm^−1^, respectively. These dates indicated that the hydrogel possessed strong adhesion ability.

**Figure 5:**
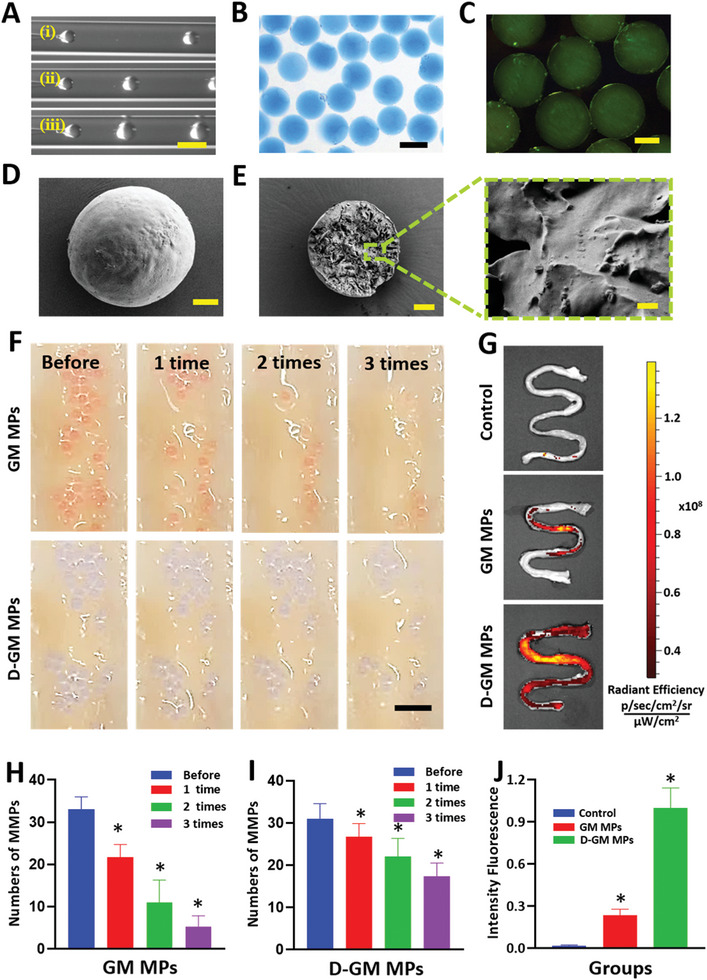
A) The real‐time images of the droplet formation procedure in the microfluidic device with outer flow rate of 200 µL min^−1^ and inner flow rate of i) 2 µL min^−1^, ii) 10 µL min^−1^, and iii) 20 µL min^−1^, respectively. Scale bar = 500 µm. B) Optical image of the microparticles dyed blue. Scale bars = 400 µm. C) Fluorescent image of the microparticles with EVs dyed green. Scale bars = 200 µm. D,E) Scanning electron microscope images of the microparticles and the inner structure. Scale bar = 50 µm. Enlarged image demonstrated the encapsulation of the EVs. Scale bar = 2 µm. F) Images of the in vitro adhesive test of the GM and D‐GM particles during three times rinsing. Scale bar = 2 mm. G) Fluorescence images of the colon received GM and D‐GM MPs. H–I) Statistic analysis of in vitro adhesive test of GM and D‐GM MPs (*n* = 4). J) Statistic analysis of the fluorescence intensity in GM and D‐GM MPs groups (*n* = 4). * 0.01 < *p* < 0.05.

To expand the application of adhesive hydrogel by rectal administration, we proposed an adhesive microparticle with EVs encapsulation via microfluidic technology. In a typical experiment, D‐GM was employed for the preparation of microparticles. During the microfluidic process, the pregel solution was pumped through the inner channel and was sheathed into micro‐droplets under the shear force of the silica oil pumped through the outer channel (**Figure** **5A**). The droplets then rapidly formed composite microparticles (D‐GM MPs) under UV radiation (Figure 5B). To examine the retention of EVs in D‐GM MPs, we encapsulated EVs (dyed with DiO) in the microparticles. As shown in Figure 5C–E, the EVs can be encapsulated in MPs. By measuring the fluorescence intensity of the supernatant of liquid containing EVs‐loaded D‐GM MPs, we found that the MPs can encapsulate EVs with almost no leakage. In addition, by regulating the flow rates of inner and outer channels, microparticles with different sizes can be achieved (Figure S8A, Supporting Information). Next, we explored the EVs loading capacity of microparticles with different sizes. Figure S9 (Supporting Information) showed that the loading capacity of microparticles with diameter of 100, 200, and 400 µm was > 95%. The release of EVs was necessary for the final curative effect, we measured the fluorescent intensity of EVs in supernatant containing microparticles with different diameters at different time points. As shown in Figure S10 (Supporting Information), MPs with smaller diameter showed higher drug release efficiency.

Furthermore, to estimate the adhesive efficacy, we adopted a strategy through in vitro attachment method to the colon epithelium of rats. First, we compared the adhesive properties of D‐GM MPs and the particles without DMA (GM MPs). As shown in Figure 5F, the GM MPs were washed away by the PBS flow easily, while the D‐GM MPs attached strongly even after rinsing for three times. As shown in Figure 5H,I, the adhesion rate of the D‐GM MPs was ≈ 18%, which was higher than that of GM MPs group, conforming that the D‐GM contributed to better adherence.

Next, the in vivo adhesive ability was conformed in IBD animal model induced by feeding dextran sulfate sodium (DSS). The rats received the same amount of D‐GM MPs and GM MPs by enema, no treatment was applied as a negative control (Control). After 12 h, these rats were sacrificed and the colons were taken out for the quantification of the fluorescence intensity. As shown in Figure 5G, there were almost no positive signal in the control group and only a few charged signals remained in the GM MPs group. However, the D‐GM MPs group had much stronger fluorescence intensity, indicating that the D‐GM MPs exhibited better adhesive performance. In addition, statistical analysis showed that the G‐DM MPs exhibited even four times more fluorescence intensity than DM MPs (Figure 5J). These results demonstrated that the adhesive microparticles could firmly adhere to the colon epidermis through rectal administration.

**Figure 6:**
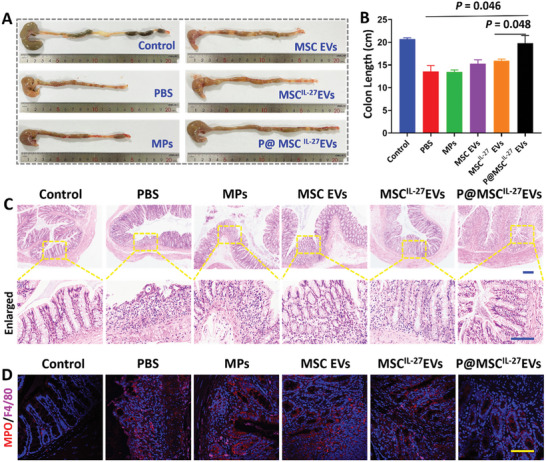
A) Images of colons length in healthy mice (Control) and IBD model treated with PBS (PBS), pure microparticles (MPs), MSC EVs, MSC^IL‐27^ EVs, and P@MSC^IL‐27^ EVs. B) Statistic analysis of the colon length from each group. (*n* = 4) C) H&E staining images of different groups（*n* = 4). Scale bar = 50 µm. D) Representative images of MPO and F4/80 immunostaining in colonic sections of different groups (*n* = 4). Scale bar = 50 µm.

In addition to testing the effective function of the IL‐27 gene modification MSC EVs‐laden D‐GM MPs in vitro, DSS induced IBD rat model was employed. First, the cell viability of Caco‐2 and RAW264.7 cells was analyzed after incubation in D‐GM MPs extracts with different concentrations (1, 5, and 10 mg mL^−1^). As shown in Figures S11 and S12 (Supporting Information), there was no obvious difference between the groups. These results confirmed the remarkable cytocompatibility of D‐GM MPs. Then, the model rats received an enema with PBS, G‐DM MPs, MSC EVs, MSC^IL‐27^ EVs, and the MSC^IL‐27^ EVs‐loaded G‐DM MPs (P@MSC^IL‐27^ EVs) on days 1 and 3, respectively, healthy rats were applied as a negative control (Control). On day 7, all rats were sacrificed and the colons were taken out for evaluation of therapeutic efficacy. The colon length was a necessary index to evaluate the curative effect. As shown in **Figure** **6**A,B, the colon length of the P@ MSC^IL‐27^ EVs group was longer than that of PBS, G‐DM MPs, MSC EVs, and MSC^IL‐27^ EVs groups, which was near to that of healthy control rats.

We further performed hematoxylin‐eosin (H&E) staining of colon samples and the main tissues to evaluate the histological status during the healing period of 7 days. As shown in Figure S13 (Supporting Information), important organ samples had no significant pathological changes. These results confirmed the remarkable long‐term in vivo biocompatibility of MPs. H&E staining image of the P@ MSC^IL‐27^ EVs group represented less inflammatory cell infiltration in lamina propria and more complete epithelial and goblet cell structure than that of PBS, G‐DM MPs, MSC EVs, and MSC^IL‐27^ EVs groups, which was near to that of the healthy rats (Figure 6C). Moreover, immunofluorescence staining also demonstrated the expression of myeloperoxidase (MPO) and F4/80, which were the biomarkers of neutrophils and mononuclear inflammatory cells, respectively. As shown in Figure 6D and Figure S14 (Supporting Information), there was a decrease of the specific indexes in P@MSC^IL‐27^ EVs group compared with the other groups.

**Figure 7:**
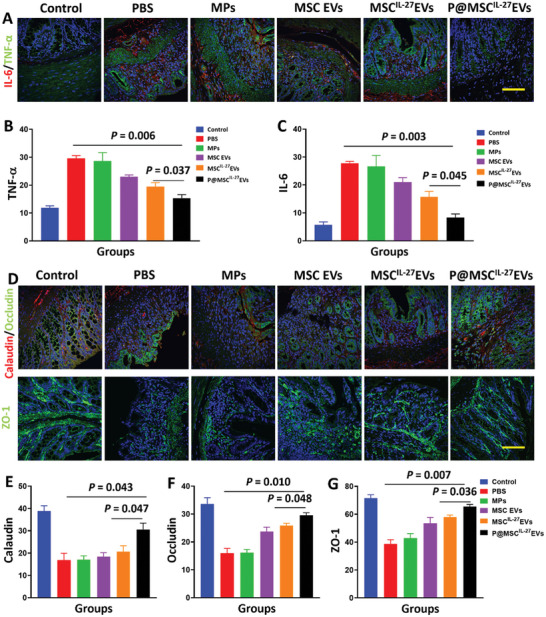
A) Representative images of IL‐6 and TNF‐*α* immunostaining in colonic sections of different groups (*n* = 4). Scale bar = 50 µm. B,C) Statistic analysis of the mean gray value from each group (*n* = 4). D) Images of calaudin, occludin, and ZO‐1 immunostaining in colonic sections of each group (*n* = 4). Scale bar = 50 µm. E,F,G) Statistic analysis of the mean gray value from different groups (*n* = 4).

To test if the anti‐inflammatory and epithelial repair generated by MSC^IL‐27^ EVs could enhance therapeutic efficacy against IBD. Inflammation‐related factors TNF‐α and IL‐6 were tested by immunofluorescence. As **Figure** **7**A–C showed, the marked reduced levels of proinflammatory factors TNF‐α and IL‐6 were found in the P@MSC^IL‐27^ EVs group. Thus, these results fully proved that the P@MSC^IL‐27^ EVs through enema delivery displayed an excellent anti‐inflammatory efficacy. Calaudin, occludin, and ZO‐1 proteins were indicators of the level of epithelial repair. We thus measured the location and relative quantification of colon samples based on immunofluorescence. Treating with P@MSC^IL‐27^ EVs led to a further trend of increase in tight junction protein including calaudin, occludin, and ZO‐1 of both precise localization and high level (Figure 7D–G). In all, our results showed P@MSC^IL‐27^ EVs treatment not only reduced inflammation‐related cytokines release but also rearranged and upregulated the expression of tight junction proteins. Therefore, P@MSC^IL‐27^ EVs could serve as an efficient therapeutic approach to treat IBD.

## 3 Conclusion

In summary, we reported IL‐27 gene modification MSC EVs‐laden adhesive microparticles for IBD treatment. MSC^IL‐27^ EVs significantly inhibited inflammation and ameliorated damage to the intestinal epithelial barrier. Furthermore, we provided an adhesive hydrogel microparticle as durable EVs carrier by using GelMA and DMA via microfluidic technology. Due to the exposure of the DOPA in DMA, the microparticles were endowed with fantastic wet adhesive property. It was demonstrated that by loading MSC^IL‐27^ EVs into these adhesive particles through rectal administration, they could firmly adhere to the colon epidermis, which contributed to an enhanced therapeutic effect and reduced the side effect for the treatment of IBD. In all, our results presented a highly accessible and biocompatible adhesive microcarriers in clinical applications of MSC derived EVs, and broadened the potential in drug delivery and the associated therapies.

## 4 Experimental Section

### Materials

The 2‐hydroxy‐2‐methylpropiophenone was obtained from Sigma–Aldrich. LPS was purchased from MedChemExpress (Monmouth Junction, NJ, USA). DSS was purchased from Yeasen Biotech. The methacrylated gelatin (GelMA) was self‐prepared. N‐[2‐(3,4‐Dihydroxyphenyl) ethyl]−2‐methylacrylamide (DMA) was purchased from Aladdin, Shanghai, China. Dulbecco's Modified Eagle Medium: Nutrient Mixture F‐12 (DMEM/F‐12), DMEM, fetal bovine serum (FBS), penicillin/streptomycin (P/S), and EDTA solution were obtained from Gibco. The Live/Dead Cell Double Staining Kit was obtained from KeyGEN BioTECH. The Cell counting kit‐8 (CCK8) assay kit was purchased from Abbkine. The GFP‐IL‐27 lentivirus was manufactured by GeneChem, Shanghai, China. IL‐27 ELISA Kit was purchased from 4A Biotech, Beijing, China. The Antibodies of IL‐27, CD81, integrin*β*, occludin, ZO‐1, F4/80, IL‐6, TNF‐*α*, and calaudin were obtained from Abcam. Anti‐MPO was obtained from Protechgroup, Wuhan, China. APC‐conjugated anti‐human CD34, PE‐conjugated anti‐human CD14, CD105, CD45, and PerCP‐Cy5.5‐conjugated anti‐human CD44 and HLA‐DR antibodies were obtained from Invitrogen, Carlsbad, Canada.

### Cell Culture

MSC performed lentivirus transfection experiment to overexpress IL‐27. Polybrene was applied to assist the viral infection, and puromycin was added for selection.

To confirm internalization of EVs, RAW 264.7, and Caco‐2 cells were incubated with 10 µg mL^−1^ EVs for 2 h. EVs were incubated with 1 mm fluorescent lipophilic tracer DiO at room temperature for 30 min prior to co‐incubated. To test the expression of related factors, Caco‐2 cells were incubated with LPS (100 ng mL^−1^) for 24 h. Next, the treated cells were co‐incubated with EVs for 24 h. After treatment, cells were collected and RNA was extracted using FastPure Cell/Tissue Total RNA Isolation Kit (Vazyme, China). The Q‐RT PCR was performed using a Fast Start Universal SYBR Green Master (Vazyme, China) in a StepOne (Thermo Fisher). To reveal the role of EVs in differentiating T cells, spleen cells were obtained from mice and grown in RPMI 1640 medium containing 10% fetal bovine serum and anti‐CD3 in 5% CO_2_ at 37 °C for 48 h. Next, the treated cells were co‐incubated with EVs and anti‐CD28 for 48 h. The related genes were detected by Q‐RT PCR. The primer sequences for Q‐RT PCR are listed in Table S1 (Supporting Information).

Live/dead cells were determined using Calcein‐AM/PI double stain kit. Images were captured by CLS. The cell viability assay was tested by co‐incubating cells with CCK‐8. The absorbance at 450 nm was measured using a multi‐model microplate reader.

### Extraction, Purification, and Identification of MSC Derived EVs with IL‐27 Overexpression

For extraction of EVs, the cells were cultured with EV‐depleted medium. The medium was centrifuged at 300× g for 10 min, 2000× g for 10 min, 10 000× g for 30 min, the collected supernatant was centrifuged at 100 000× g for 70 min, the supernatant was removed and PBS was used to resuspend the pellet, 100 000× g for 70 min at 4 °C to obtain MSC EVs. TEM was applied to identify the spherical morphology and diameter of EVs. The size distribution of EVs was measured by DLS.

### Fabrication and Characterization of G‐DM Hydrogel

In brief, 0.1 g DMA was dissolved in 400 µl dimethyl formamide (DMF) under magnetic stirring at room temperature (RT). After DMA was dissolved, 600 µl distilled water, 0.1 g GelMA, and 20 µL HMPP were added to dissolve. The pregel was exposed to the UV light for 90 s to form G‐DM hydrogel. To load the EVs, 1 ml pregel was mixed with 10 mg EVs.

The adhesive, shear and peel strength of G‐DM hydrogel on porcine skin were observed by the standard tensile test (ASTM F2258), shear test (ASTM F2255), and peel test (ASTM F2256), respectively, using tensile machine (INSTRON, Germany).

### Preparation of EVs‐Loaded G‐DM Microparticles

To fabricate EVs‐loaded G‐DM microparticles, we first established a double emulsion microfluidic chip. Two capillaries with inner diameter of 580 µm were applied. The orifice of one of the capillaries was tapered to a diameter of ≈ 200 µm, and inserted into the other capillary coaxially on a glass slide. To fix the two capillaries and facilitate the inner and outer phase pumping through, two syringe needles were applied. EVs were then suspended in the G‐DM pregel solution, which was subsequently pumped through as the inner phase using a syringe pump (PHD 2000, Harvard Apparatus). In the meantime, silica oil was pumped through as the outer phase to shear the G‐DM inner phase into micro‐droplets. When the micro‐droplets were collected in a dish containing silica oil, UV light was employed to solidify the EVs‐loaded G‐DM micro‐droplets to achieve EVs‐loaded G‐DM microparticles. Then, the extra silica oil was removed by PBS. Finally, the obtained EVs‐loaded G‐DM microparticles were kept in PBS at room temperature in darkness for downstream experiments.

### The Release Test of EVs

EVs were incubated with 1 mm DiO at RT for 30 min before fabricating microparticles. A spectrometer detected the supernatant at 488 nm to measure the concentration of EVs in different diameters of microparticles, at different time points.

### In Vitro and In Vivo Microparticles Adhesion Experiments

For in vitro attachment test, a drop of PBS buffer containing GM and D‐GM MPs were rinsed through the colon epithelium of rats, respectively. After that, 1 mL of PBS was applied to wash it for another three times.

For in vivo adhesion experiments, the IBD model rats received an enema with GM and D‐GM MPs were used for the adhesion experiment. The rats were starved overnight and received 500 µL the same amount of microparticles at the following morning by enema. After 12 h, the rats were sacrificed and the colon was removed and imaged immediately. EVs were incubated with 1 mM DiO at RT for 30 min prior to enema. The fluorescence signal intensity was monitored using in vivo imaging system.

### Animals

All animal care and handing procedures were conformed with the ethics (ethics number 2020AE02014). The IBD model was established by feeding 5% DSS for 10 days. Then, the DSS modeling rats received an enema with PBS, G‐DM MPs, MSC EVs, MSC^IL‐27^ EVs, and P@ MSC^IL‐27^ EVs on days 1 and 3, respectively, healthy rats were applied as a negative control (Control). On day 7, the rats were sacrificed, and removed the colons. After colons isolation and fixation in paraformaldehyde, the tissues were embedded in paraffin for H&E and immunofluorescence experiments. The tissues were cut for 7 µm for immunohistochemistry according to the manufacturer's instructions.

### Statistical Analysis

Graph Pad Prism was used for graphing and statistical analysis. Significant differences between groups were checked using two‐tailed Student's *t* test (comparing two groups) or one‐way ANOVA followed by Tukey post‐hoc test in multiple comparisons, as indicated in the figure legend. All quantification data were presented as mean values ± SEM. Experiments have at least three independent replicates.

## Conflict of Interest

The authors declare no conflict of interest.

## Supporting information

Supporting Information

## Data Availability

The data that support the findings of this study are available in the supplementary material of this article.
