# Supplementary material for: Bioadhesive Microcarriers Encapsulated with IL‐27 High Expressive MSC Extracellular Vesicles for Inflammatory Bowel Disease Treatment
Source: Adv Sci (Weinh). 2023 Sep 27;10(32):2303349. doi: 10.1002/advs.202303349 (PMC10646269; doi:10.1002/advs.202303349)
Supplement: Supplementary file 1 — Supporting Information [file ADVS-10-2303349-s001.pdf]

ADVANCED SCIENCE
Open Access

Supporting Information

for *Adv. Sci.*, DOI 10.1002/advs.202303349

Bioadhesive Microcarriers Encapsulated with IL-27 High Expressive MSC Extracellular Vesicles for Inflammatory Bowel Disease Treatment

Min Nie, Danqing Huang, Guopu Chen, Yuanjin Zhao and Lingyun Sun**

Supporting information

Bioadhesive microcarriers encapsulated with IL-27 high expressive MSC extracellular vesicles for inflammatory bowel disease treatment

Min Nie, Danqing Huang, Guopu Chen, Yuanjin Zhao, Lingyun Sun**

M. Nie, D. Q. Huang, G. P. Chen, Prof. Y. J. Zhao, Prof. L. Y. Sun
Department of Rheumatology and Immunology, Nanjing Drum Tower Hospital, Affiliated Hospital of Medical School, Nanjing University, Nanjing 210002, China

Email: lingyunsun@nju.edu.cn (L. Y. Sun); yjzhao@seu.edu.cn (Y. J. Zhao)

Prof. L. Y. Sun
Department of Rheumatology and Immunology, The First Affiliated Hospital of Anhui Medical University, Hefei 230022, China

Prof. Y. J. Zhao
State Key Laboratory of Bioelectronics, School of Biological Science and Medical Engineering, Southeast University, Nanjing 210096, China

Table S1. List of individual gene primers.

Gene	Forward	Reverse
Human-IL-27	TGCCAGGAGTGAACCTGTACC	CGTGGTGGAGATGAAGCAGA
Human-TLR4	GTACCTGGGGAACAACCTCTT	GCAGCTTGACTAGACTCTCCA
Human-TNF-α	CCCAGGCAGTCAGATCATCTTC	AGCTGCCCCTCAGCTTGA
Human-IL-6	GACAGCCACTCACCTCTTCA	CCTCTTTGCTGCTTTCACAC
Human-IL-8	GCCAACACAGAAATTATTGTAAAGCTT	AATTCTCAGCCCTCTTCAAAAACTT
Human-ZO-1	GAGCCTAATCTGACCTATGAACC	TGAGGACTCGTATCTGTATGTGG
Human-occludin	CTTCCAATGGCAAAGTGAATG	TACCACCGCTGCTGTAACGAG
Mouse-IL-10	GCTGGACAACATACTGCTAACC	ATTTCCGATAAGGCTTGGCAA
Mouse-IL-4	CCCCAGCTAGTTGTCATCCTG	CAAGTGATTTTTGTCGCATCCG
Mouse-IFN-γ	GCCACGGCACAGTCATTGA	TGCTGATGGCCTGATTGTCTT
Human-GAPDH	CGAGCCACATCGCTCAGACA	GTGGTGAAGACGCCAGTGGA

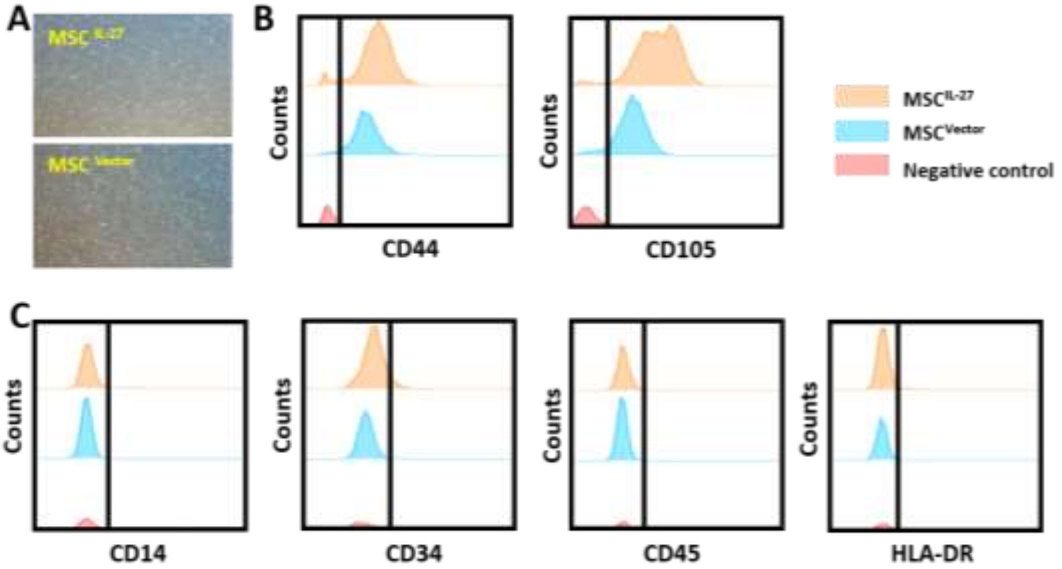

Figure S1. Characteristics of MSC^{IL-27} and MSC^{Vector}. (A) Representative optical morphological images of MSC^{IL-27} and MSC^{Vector}. (B) Flow cytometric analysis showed MSC^{IL-27} and MSC^{Vector} were positive for CD44 and CD105. (C) Flow cytometric analysis showed MSC^{IL-27} and MSC^{Vector} were negative for CD14, CD34, CD45 and HLA-DR.

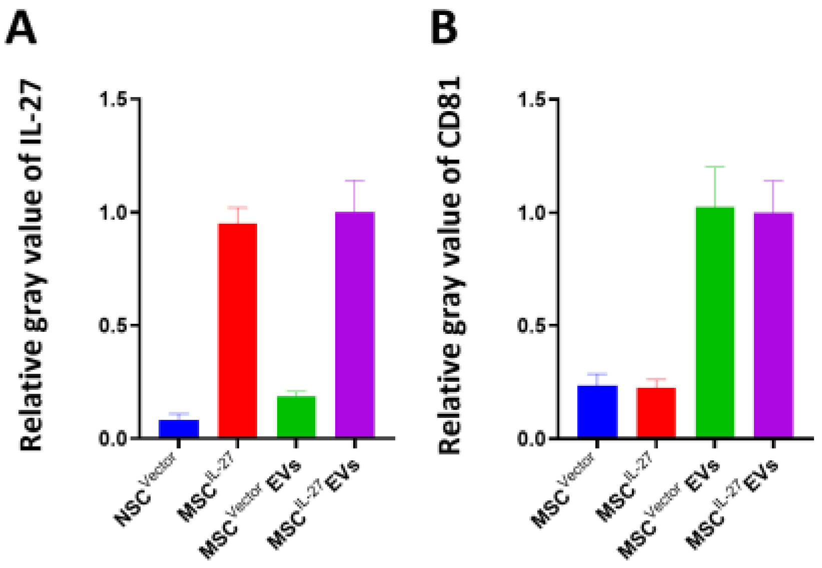

Figure S2. Gray values of IL-27 and CD81 to GAPDH protein as interior reference were relative amounts of IL-27 (A) and CD81 (B).

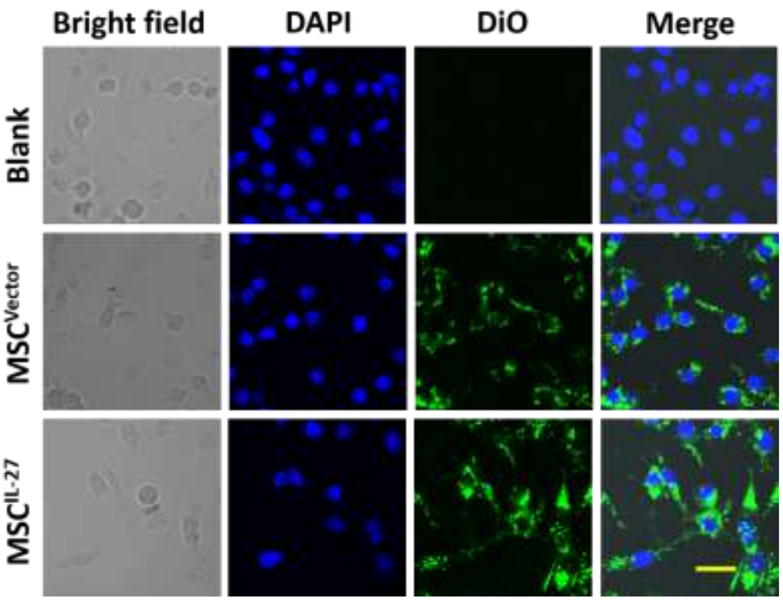

Figure S3. CLSM of RAW 264.7 cells incubated with DiO labeled EVs. Scale bars = 100 μm.

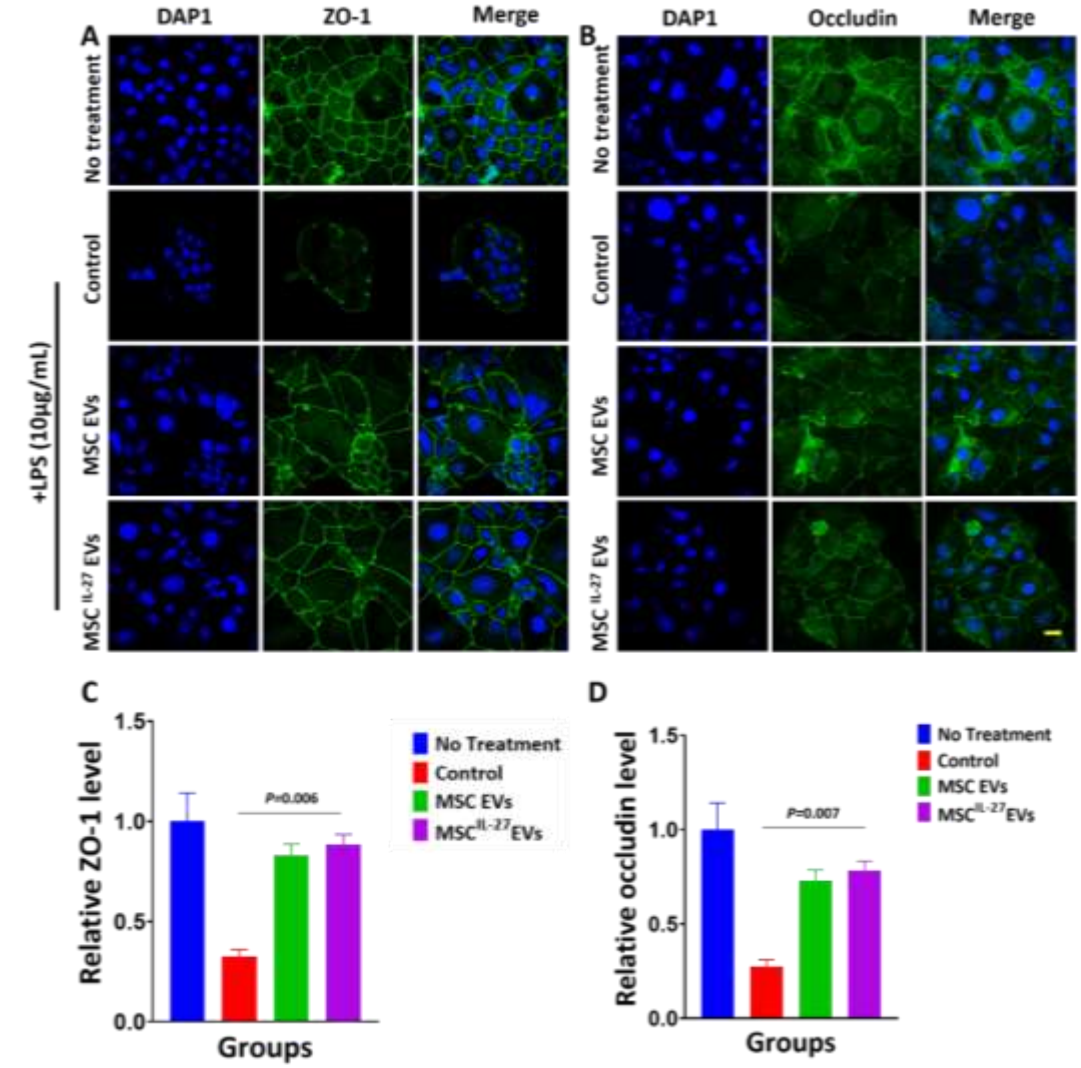

Figure S4. ZO-1 (A) and occluding (B) expressions were analyzed by CLSM. Scale bars=100μm. (C, D) Quantitative measurement of immunofluorescent staining of ZO-1 and occludin.

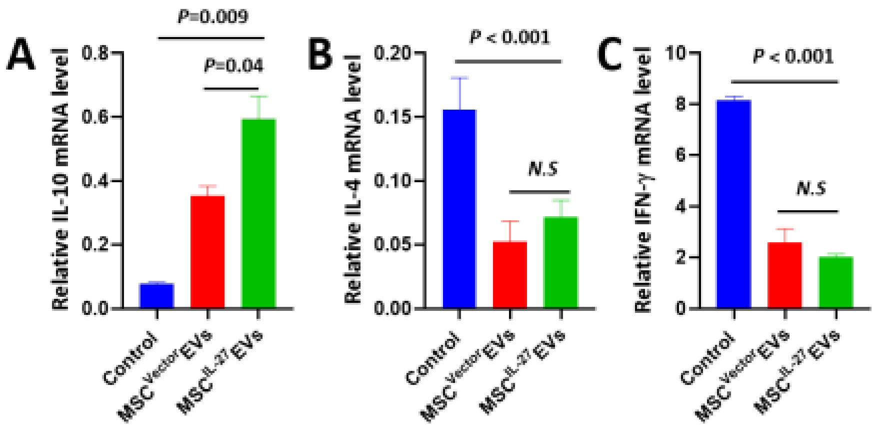

Figure S5. IL-10 (A), IL-4 (B) and IFN-γ (C) expressions were analyzed by Q-RT PCR. N.S: no significance.

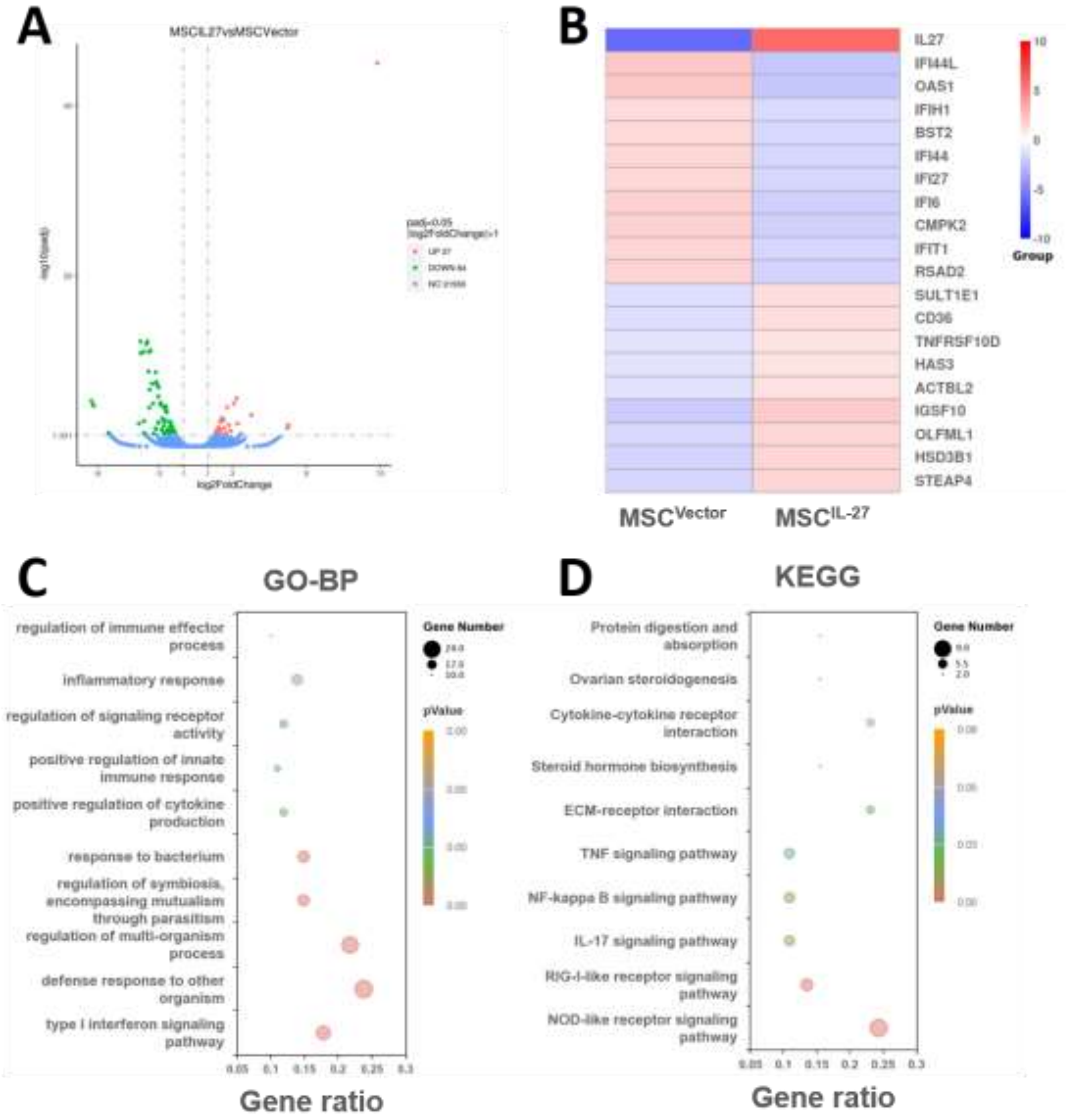

Figure S6. (A) Differential gene statistics. The abscissa was Log2 Fold Change value, the ordinate was -Log10 (*p* value), and the dashed blue line represented the threshold line of the differential gene screening criteria. (B) The expression of marker genes in MSC^{Vector} and MSC^{IL-27} groups. (C) GO analysis of top 10 different terms related to biological processes (BP) in MSC^{IL-27} compared to MSC^{Vector} groups. (D) KEGG analysis of top 10 different terms in MSC^{IL-27} compared to MSC^{Vector} groups.

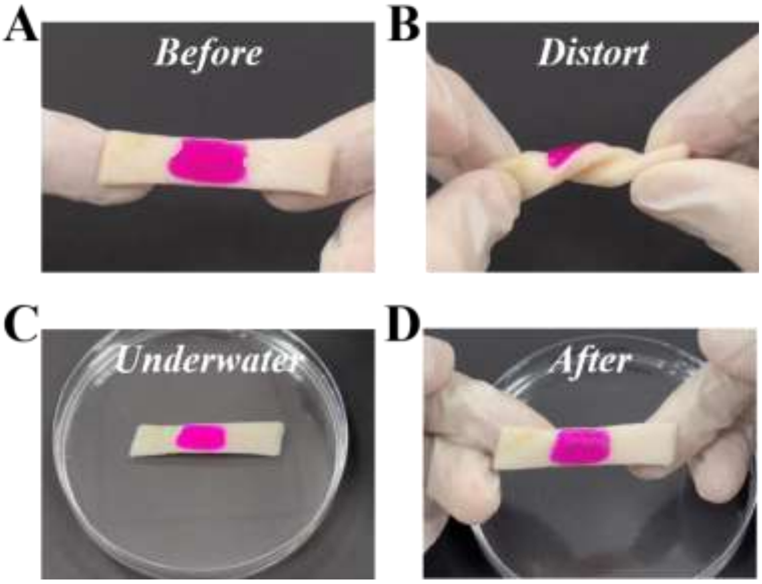

Figure S7. Setups for mechanical testing of adhesion performance.

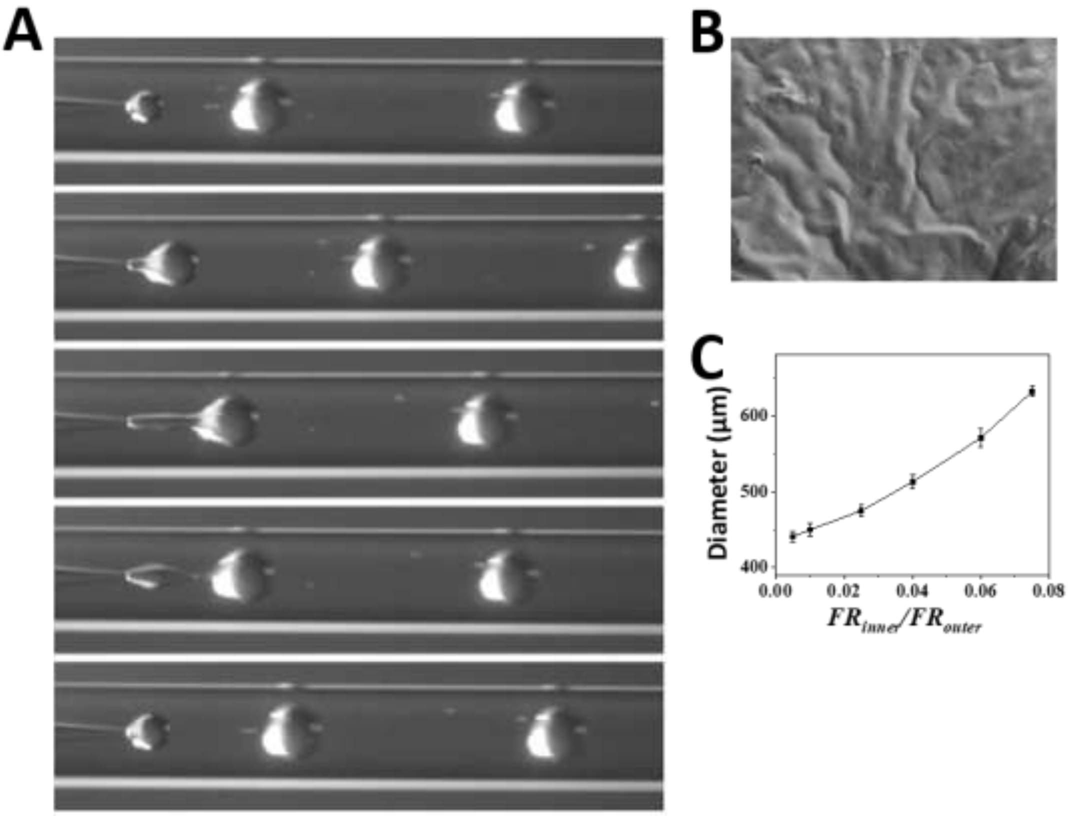

Figure S8. (A) The real time generation images of the formation of the D-GM microparticles from co-flow geometry microfluidics. (B) SEM images of the resultant microparticles. (C) Relationships of the diameters of the microparticles with the FR_{inner}/FR_{outer}.

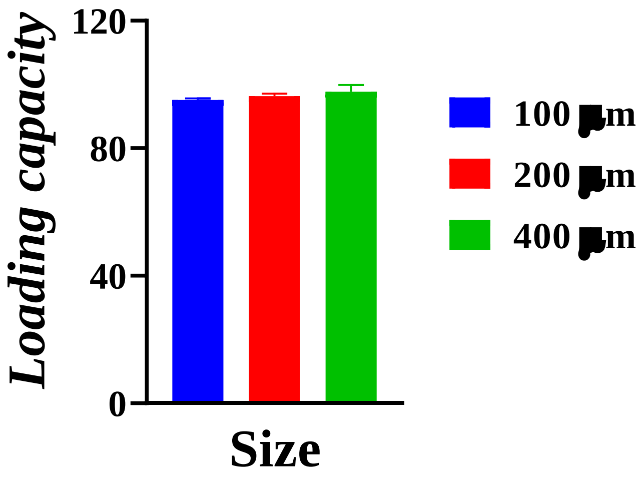

Figure S9. The loading capacity of microparticles with different sizes.

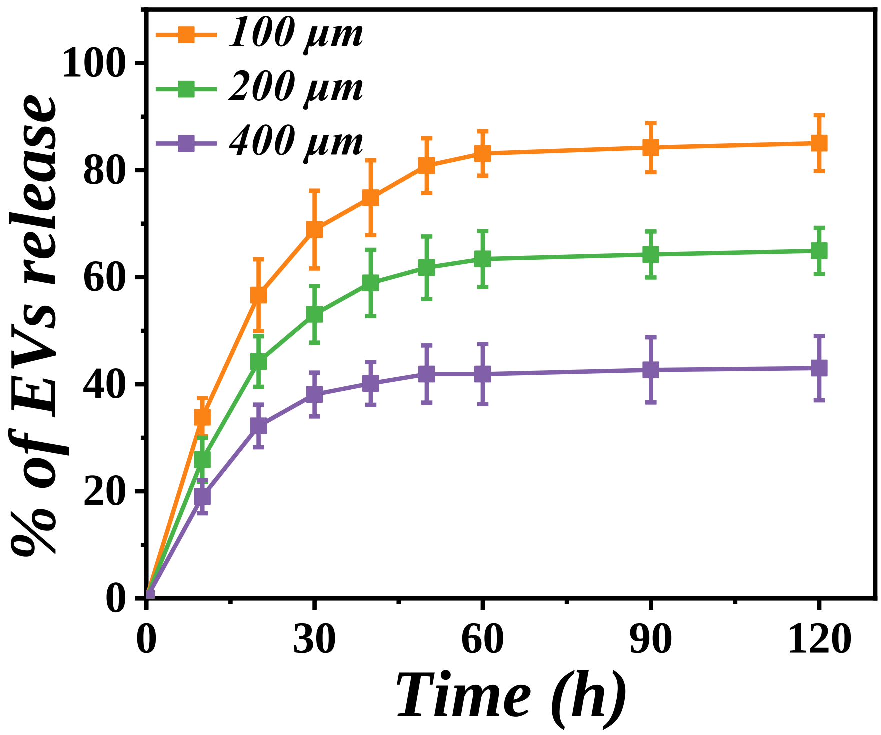

Figure S10. The release rate of microparticles with different diameters at different time points.

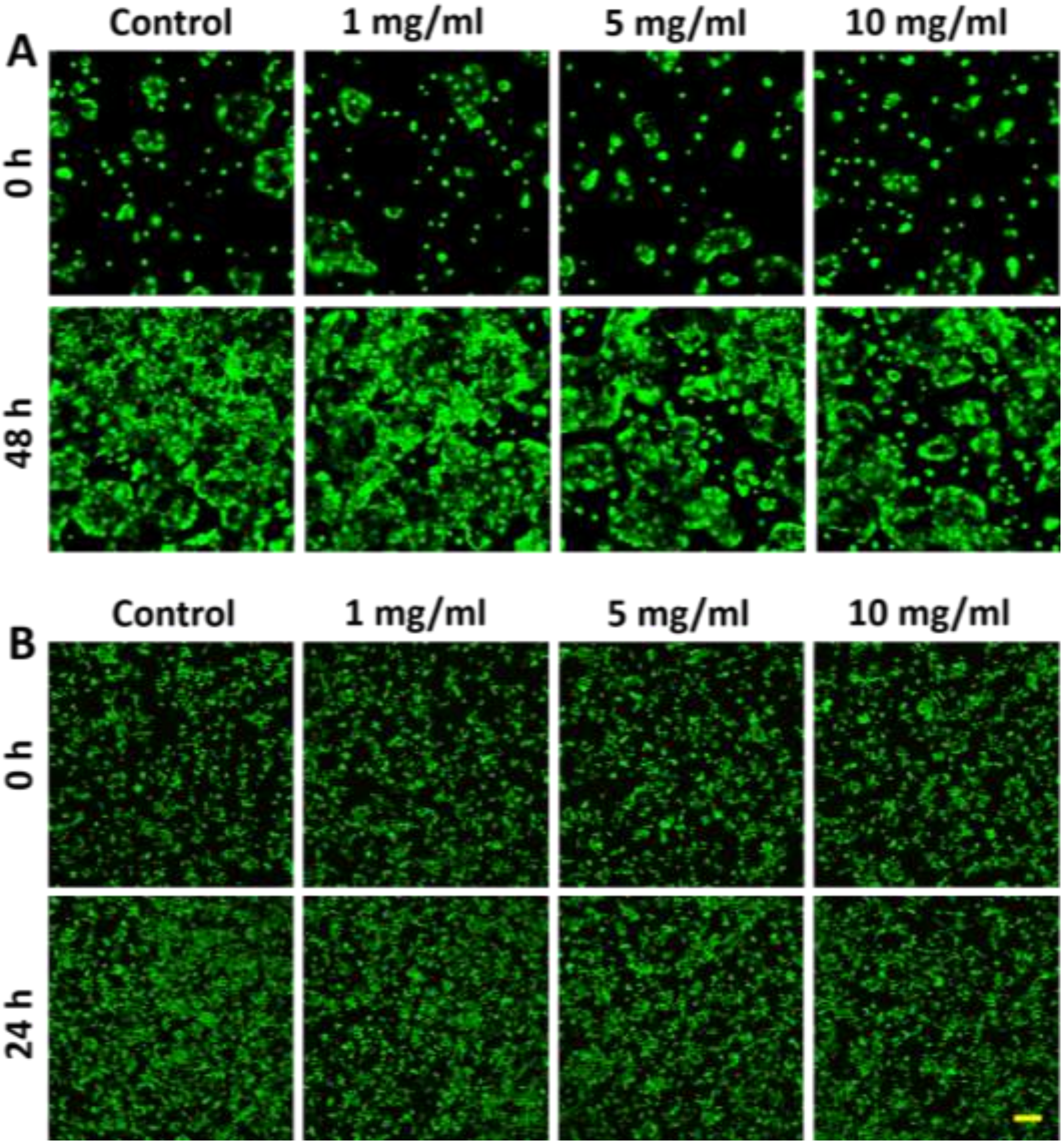

Figure S11. Cytocompatibility evolution of the hydrogels extracts against Caco-2 (A) and RAW 264.7 (B) cells with different concentrations. Scale bars=100μm.

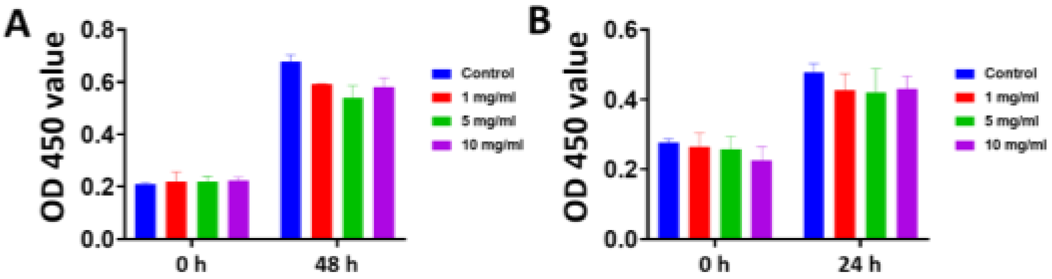

Figure S12. The viability of Caco-2 (A) and RAW 264.7 (B) cells exposed to hydrogel extracts with different concentrations was evaluated using CCK-8 assay.

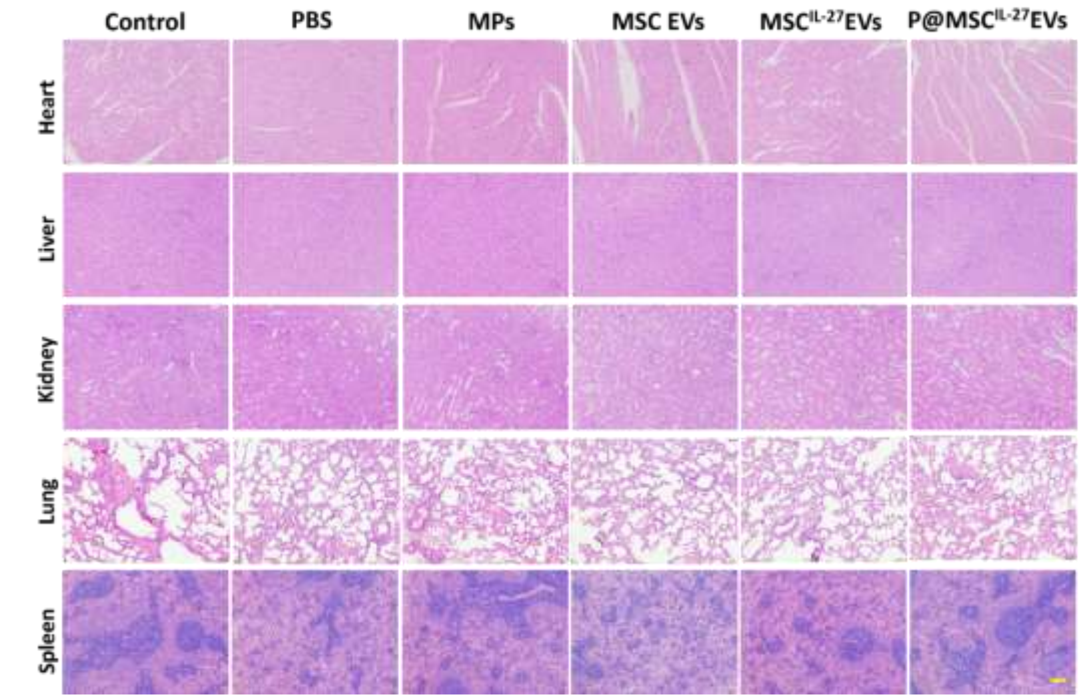

Figure S13. H&E stained organs revealed no significant signs of toxicity. The scale bar is 100 μm.

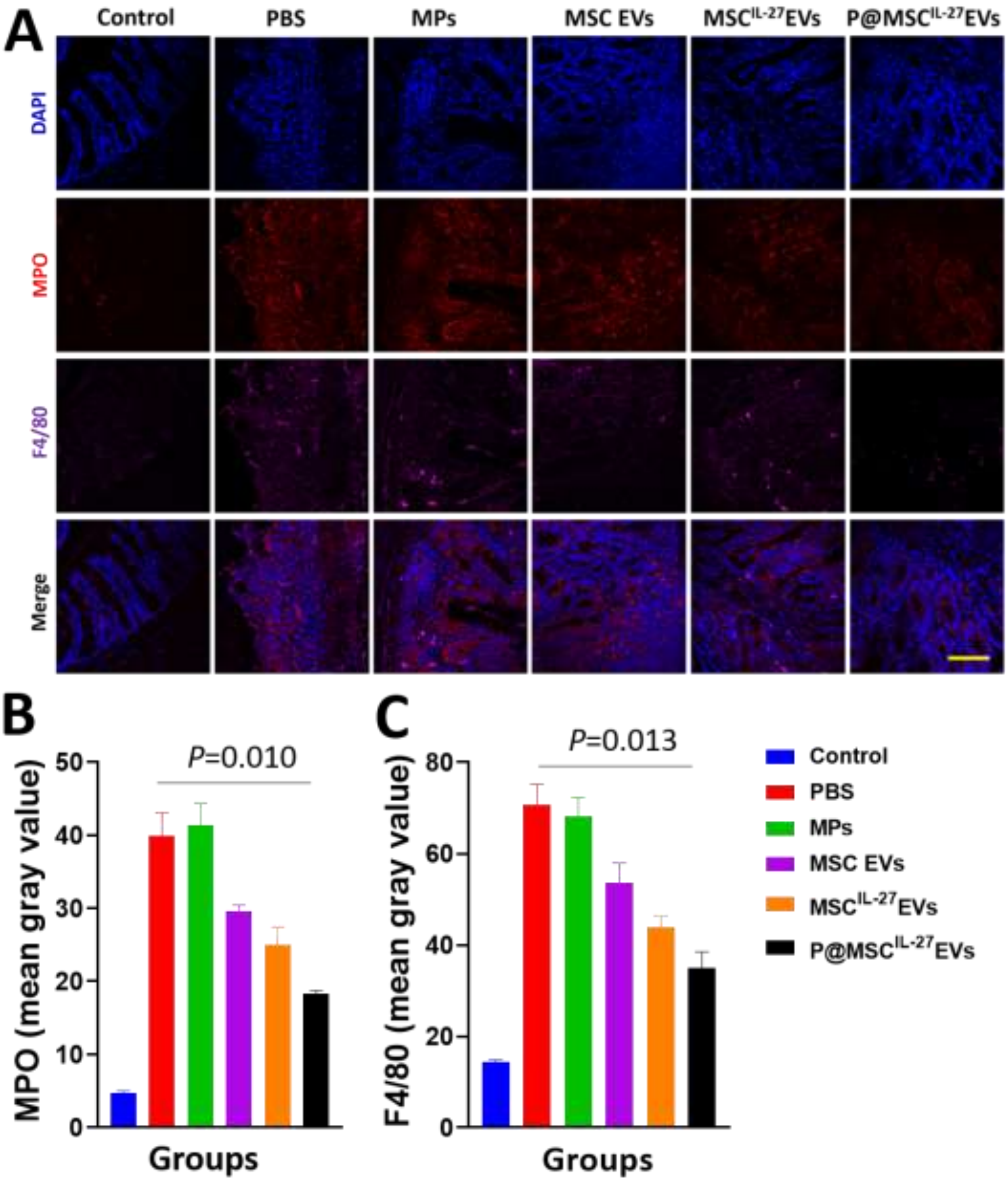

Figure S14. (A) Representative images of MPO and F4/80 immunostaining in colonic sections of different groups. Scale bar = 50 μm. (B) The mean gray value of MPO and F4/80 from different groups.
